# Therapeutic use of α2-antiplasmin as an antifibrinolytic and hemostatic agent in surgery and regenerative medicine

**DOI:** 10.1038/s41536-022-00230-x

**Published:** 2022-06-30

**Authors:** Jialu Liu, Ani Solanki, Michael J. V. White, Jeffrey A. Hubbell, Priscilla S. Briquez

**Affiliations:** 1 Pritzker School of Molecular Engineering, University of Chicago, Chicago, IL USA; 2 Animal Resources Center, University of Chicago, Chicago, IL USA; 3 Committee on Immunology, University of Chicago, Chicago, IL USA; 4 Committee on Cancer Biology, University of Chicago, Chicago, IL USA; 5 Department of General and Visceral Surgery, Medical Center - University of Freiburg, Freiburg, Germany

**Keywords:** Regenerative medicine, Translational research

## Abstract

The biomaterial fibrin is widely used as a clinical tissue sealant in surgery. In preclinical research, fibrin is also extensively studied as a carrier material for growth factor delivery. In these applications, premature fibrin degradation leads to recurrent bleeding, tissue dehiscence and limited regenerative efficacy. Therefore, fibrinolysis inhibitors have been added to clinical fibrin formulations, for example the bovine-derived serine protease inhibitor aprotinin. Aprotinin is additionally used as a hemostatic agent to prevent excessive bleeding during surgery, in this case protecting endogenous fibrin clots. Nevertheless, aprotinin use has been associated with serious safety issues. Here, we explore the use the human physiological fibrinolysis inhibitor α2-antiplasmin (α2PI) as a substitute for aprotinin. We evaluate the efficacy of α2PI in the three main applications of aprotinin. We first showed that recombinant α2PI can successfully prolong the durability of fibrin biomaterials as compared to aprotinin in a model of subcutaneous implantation in mice mimicking application as a tissue sealant. We then used α2PI to enhance the delivery of engineered vascular endothelial growth factor (VEGF)-A and platelet-derived growth factor (PDGF)-BB in fibrin in promoting diabetic wound healing, which lead to improved wound closure, granulation tissue formation and angiogenesis. Lastly, we demonstrated that α2PI can be as effective as aprotinin as an intravenous hemostatic agent to prevent blood loss, using a tail-vein bleeding model in mice. Therefore, we believe that engineering fibrin biomaterials or endogenous fibrin with α2PI can have a strong impact in surgery and regenerative medicine by providing a competitive substitute to aprotinin that is of human origin.

## Introduction

Fibrin plays a central role in hemostasis and during wound healing as being a main component of blood clots^1^. In the clinic, fibrin biomaterials are widely used as tissue sealants to stop hemorrhages and reattach tissues during surgeries^2,3^. In addition, fibrin is extensively explored as a carrier material for drug delivery and as a cell scaffold in tissue engineering research^4–7^. For example, growth factors (GFs), such as vascular endothelial growth factor (VEGF)-A or platelet-derived growth factor (PDGF)-BB, have been successfully delivered via fibrin hydrogels to accelerate diabetic wound healing^7^.

Currently, clinical-grade fibrin is made from human fibrinogen purified from blood donors, which limits its sourcing, generates batch-to-batch variability and is associated to a risk of pathogen transmission^8^. Moreover, clinical fibrin needs to be applied at very high dose of 22.5–45.5 mg/mL to provide appropriate strength to ensure good tissue sealing and counteract its rapid degradation upon delivery, which further reduces its cost-effectiveness^9,10^. Indeed, the inflamed tissues in which fibrin is applied, such as acute surgical wounds or chronic diabetic wounds, are characterized by increased levels of fibrinolytic proteases that prematurely degrade fibrin, thus leading to unwanted bleeding, tissue dehiscence and suboptimal drug delivery.

In addition to high doses, fibrin stability has been improved by the incorporation of protease inhibitors in some clinical formulations, for example of aprotinin in TISSEEL^®^ (Baxter)^11^. Aprotinin is a clinically approved bovine-derived broad spectrum serine protease inhibitor and remains to date one of the most effective anti-fibrinolytic agents. In addition to use in fibrin sealants, aprotinin is used as a hemostatic agent to prevent excessive bleeding during surgeries. Indeed, perioperative intravenous injection of aprotinin, marketed under the product Trasylol^®^ (Bayer Pharmaceuticals), reduces the need for blood transfusions during coronary artery bypass graft surgery (CABG), thoracic surgery, liver transplantation and orthopedic surgery^12,13^. Aprotinin is commonly injected at a 140–280 mg loading dose followed by 35–70 mg/hr constant infusion during these surgeries^12^. In these applications, aprotinin protects endogenous fibrin clots from premature degradation, therefore improving patient blood coagulation.

Despite its potent efficacy, aprotinin’s safety remains a serious concern, particularly when administered intravenously. The side-effects of aprotinin include adverse immune reactions due to the bovine origin of the drug, ranging from skin rashes to severe anaphylactic shock, particularly upon drug re-exposure^14–16^. Additional renal and cardiovascular toxicities have been reported, leading to an increased risk of death. Even topical use of aprotinin could lead to development of aprotinin-specific antibodies, which poses risks for anaphylactic reactions upon any form of re-exposure^15^. Due to these safety concerns, Trasylol^®^ received a warning from the U.S. Food and Drug Administration (FDA) in 2006 and was withdrawn from hospitals and pharmacies in 2008^17^, although its use remains possible under a special request as an investigational drug.

**Fig. 1:**
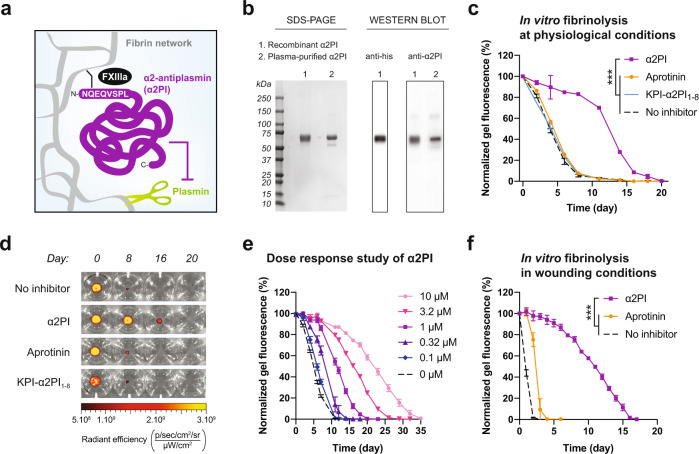
Recombinant human α2PI protects fibrin biomaterials more effectively than the clinical protease inhibitor aprotinin. **a** Schematic view of α2PI enzymatically crosslinked into fibrin by Factor XIIIa (FXIIIa) at its N-terminus and inhibiting plasmin-mediated fibrinolysis. **b** SDS-PAGE and western blot analysis of recombinant α2PI, under reducing conditions, as compared to the commercially available α2PI purified from human plasma. **c** Degradation of fibrin containing 1 μM of α2PI, aprotinin, KPI-α2PI_1–8_ or no inhibitor in presence of 2.5 nM of plasmin. Fibrinolysis is monitored via reduction in fibrin fluorescence over time. **d** Representative images of the degradation of fibrin gels containing the different inhibitors, as presented in **c**. **e** Efficacy of α2PI in protecting fibrin biomaterials at different concentrations ranging from 0 to 10 μM, in the presence of 2.5 nM of plasmin. **f** Efficacy of α2PI (15 μM) to slow fibrinolysis in the presence of 25 nM of plasmin, mimicking the increased activity of plasmin in wounding conditions. Mean ± standard deviation (SD). Statistical comparisons were done using ANOVA test with Dunnett’s post-hoc test: ***p-value < 0.001.

In this study, we explore the therapeutic use of α2-antiplasmin (α2PI) as a potential substitute to aprotinin for local and systemic applications. α2PI is the main inhibitor of plasmin in humans, with plasmin being one of the most important fibrinolytic protease^18^. Interestingly, α2PI naturally crosslinks into fibrin during clotting due to the presence of a transglutaminase substrate sequence (α2PI_1–8_) at its N-terminus, which could sustain its antifibrinolytic effects by preventing its rapid release from fibrin (Fig. 1a). This, along with the human origin of α2PI, might constitute strong advantages over aprotinin. We first evaluate the efficacy of α2PI in protecting subcutaneous fibrin implants, modeling an application in use in fibrin sealants. We then examine α2PI as a fibrin supplement for the delivery of GFs in diabetic wound healing, particularly delivering engineered variants of VEGF-A and PDGF-BB that display super-affinity to the extracellular matrix^19^. Lastly, we explore the use of systemic α2PI as a hemostatic agent for bleeding reduction during surgery.

## Results

### Expression of recombinant α2PI in mammalian cells

Recombinant expression of therapeutic proteins represents a strong advantage for future clinical translation. Therefore, we started by assessing the recombinant expression of his-tagged human α2PI upon transient transfection in HEK293-F mammalian cells. After 7 days in culture, the supernatant was purified by Ni^2+^-based immobilized metal ion affinity chromatography and analyzed by SDS-PAGE and Western blot analysis, using both anti-his-tag and anti-α2PI detection antibodies. These analyses revealed the presence of pure recombinant α2PI at similar molecular weight than the plasma-purified α2PI, purchased commercially (Fig. 1b). In total, 22.1 ± 3.1 mg of pure α2PI was obtained per liter transfection (mean of 3 independent production batches). We then confirmed the bioactivity of recombinant α2PI in preventing fibrinolysis in vitro. To do so, we incorporated α2PI into fluorescently-labeled fibrin gels during polymerization, incubated them with plasmin and monitored fibrin degradation via fluorescence decay. The results demonstrated that recombinant α2PI had similar inhibitory efficacy on plasmin as the plasma-purified α2PI (Fig. S1).

### In vitro protection of fibrin biomaterial by α2PI

We then evaluated the effectiveness of α2PI in extending fibrin longevity in vitro as compared to the clinically used aprotinin, comparing as well as to the engineered inhibitor KPI-α2PI_1–8_ previously developed in our laboratory and made by the fusion of the transglutaminase substrate domain α2PI_1–8_ and a human Kunitz-type protease inhibitor domain (KPI)^20^. We incorporated the different protease inhibitors into fluorescent fibrin gels (10 mg/mL of fibrinogen), using a concentration of 1 μM of inhibitors, which corresponds to the physiological concentration of α2PI in blood^21,22^. The fibrin gels were incubated in a buffer containing 2.5 nM of plasmin, also mimicking physiological level of plasmin in plasma, to assess gel degradation over time^23^. We found that the fibrin gels supplemented with aprotinin or KPI-α2PI_1–8_ did not stay longer than fibrin gels without inhibitor, all of them lasting for about 8 days. In contrast, α2PI significantly prolonged fibrin longevity to about 19 days, which represents a 2.3x extension (Fig. 1c, d). Of note, we previously showed that higher doses of aprotinin and KPI-α2PI_1–8_ can prolong the longevity of fibrin gels when used at a higher concentration of 15 μM in a similar assay^20^. In addition, we confirmed that our experimental results were not biased by the release of unconjugated fluorescent molecules from non-degraded fibrin gels; indeed, we obtained similar results when measuring the reduction in diameter of non-fluorescent fibrin gels over time, when using the same inhibitor formulations and under the same experimental conditions (Fig. S2a, S2b).

Next, we performed a dose-response study of α2PI in vitro to optimize fibrin longevity prior to in vivo experimentation. We kept a physiological concentration of plasmin and tested 5 different concentrations of α2PI, ranging from 0.1 μM to 10 μM, incorporated in fibrin gels (made of 10 mg/mL of fibrinogen). As expected, we showed that the higher the α2PI concentration, the slower was the fibrin gel degradation (Fig. 1e), with an almost linear relation between the gel duration and the logarithm of α2PI concentration (Fig. S2c). Moreover, we noticed that the effectiveness of α2PI did not reach a plateau at the highest tested dose of 10 μM, suggesting that an additional increase in dose would further prolong fibrin duration in vitro.

Considering the overexpression of plasmin in many applications of regenerative medicine, such as during wound healing and particularly in diabetic wounds^24–26^, we repeated the in vitro fibrinolysis assay using a 10-fold increased concentration of plasmin (25 nM) and 15 μM of α2PI, aprotinin or no inhibitor in fibrin gels (10 mg/mL of fibrinogen). In such a high proteolytic environment, α2PI vastly outperformed aprotinin, protecting fibrin gels for about 16 days as compared to 4 days for aprotinin, and 2 days for fibrin in absence of inhibitor (Fig. 1f).

### α2PI-mediated protection of fibrin sealant in vivo at low fibrinogen concentrations

**Fig. 2:**
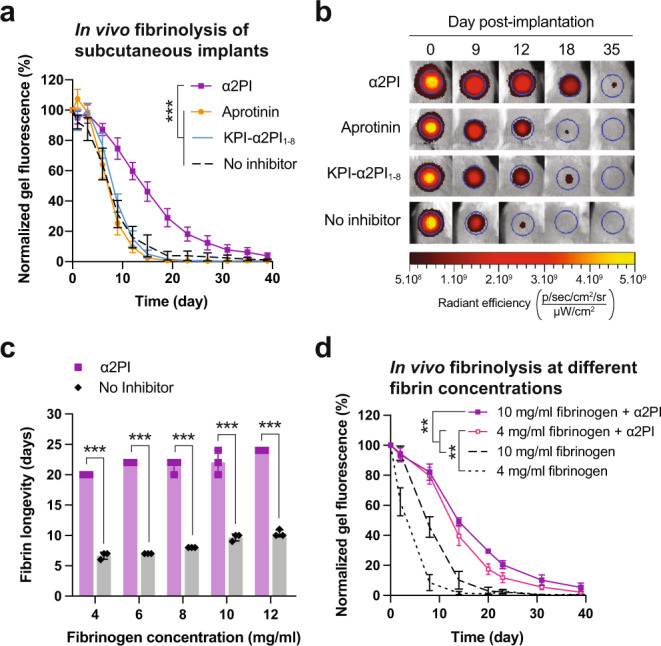
In vivo efficacy of α2PI to protect fibrin biomaterials as compared to the clinical aprotinin and at low-concentration fibrin. **a** Degradation of fibrin gels containing 15 μM of α2PI, aprotinin, KPI-α2PI_1–8_ or no inhibitors when implanted subcutaneously in the back of mice (*n* = 5 gels/group). Fibrin degradation was monitored via fluorescence reduction quantified by IVIS imaging. **b** Representative images of the degradation of the subcutaneous fibrin implants, as presented in **a**. **c** In vitro longevity of fibrin gel supplemented with α2PI (1 μM) or without inhibitor according to the concentration of fibrinogen when incubated with 2.5 nM plasmin. Mean ± SD. Statistical comparison was done using Student’s t-test. **d** In vivo degradation of fibrin gels made of 4 mg/mL or 10 mg/mL of fibrinogen containing 15 μM α2PI or no inhibitor, when implanted subcutaneously in the back of mice (*n* = 5 gels/group). Mean ± standard error of the mean (SEM) if not stated otherwise. Statistical comparisons were done using ANOVA test with Dunnett’s post-hoc test if not stated otherwise: **p-value < 0.01, ***p-value < 0.001.

We then examined the durability of α2PI-containing fibrin gels upon subcutaneous implantation in the back of mice, assessing its potential for application in fibrin sealants. Therefore, we compared the degradation time of fluorescently-labeled fibrin gels (10 mg/mL of fibrinogen) containing 15 μM of α2PI, aprotinin or KPI-α2PI_1–8_. While fibrin gels containing aprotinin or KPI-α2PI_1–8_ degraded between day 18 and 23, similarly to fibrin with no inhibitor, α2PI significantly extended fibrin gel durability to about 40 days (Fig. 2a, b), thus doubling fibrin duration.

Considering the strong efficacy of α2PI in prolonging the presence of fibrin in vivo, we asked whether the use of α2PI could allow for a reduction in fibrinogen concentration in clinical fibrin sealants. Indeed, fibrinogen has limited sourcing, a relatively high cost, and is used at high concentration in part to counteract its fast degradation upon implantation. Therefore, we assessed the degradation time of fluorescent fibrin gels made from 5 different concentrations of fibrinogen, ranging from 4 to 12 mg/mL, supplemented with 1 μM of α2PI. Interestingly, we found that reducing fibrin concentration had only minimal effects on the gel durability; indeed, a 3x reduction in concentration from 12 to 4 mg/mL fibrinogen reduced fibrin duration from 24 to 20 days when supplemented with α2PI, yet from 10 to 6.5 days in absence of inhibitor (Fig. 2c). Importantly, this highlighted the ability of α2PI to protect fibrin biomaterials formed at low fibrin concentrations.

Based on this result, we assessed the potential of α2PI to protect low-dose fibrin biomaterials in vivo, in the subcutaneous implantation model in mice, using a dose of 15 μM α2PI. In the absence of inhibitor, the 4 mg/mL fibrin gels degraded in about 10 days, which is about 50% faster than the 10 mg/ml fibrin gels, which lasted about 20 days (Fig. 2d, Fig. S3). In contrast, both the 4 and 10 mg/ml fibrin gels containing α2PI degraded much slower, lasting around 35 and 40 days for the 4 and 10 mg/mL gels respectively.

### α2PI-mediated protection of fibrin in diabetic wounds

In addition to use in tissue sealants, fibrin has been extensively used in pre-clinical research as a carrier material for drug delivery, particularly for the delivery of GFs to enhance diabetic wound healing. Here, we studied the effect of α2PI-supplemented fibrin for application in wound healing in the db/db mouse model of type II diabetes. We confirmed that, upon wounding, the concentration of plasminogen significantly increased in the wound at day 3 and returned close to baseline after day 7 (Fig. S4). Of note, others have shown that plasminogen levels in the wounds of diabetic db/db mice remained overall lower than in the non-diabetic db/+ mice (about 0.9 ± 0.05 μg/mL plasminogen in db/db mice vs. 3.6 ± 0.3 μg/mL in db/+ mice)^27^.

**Fig. 3:**
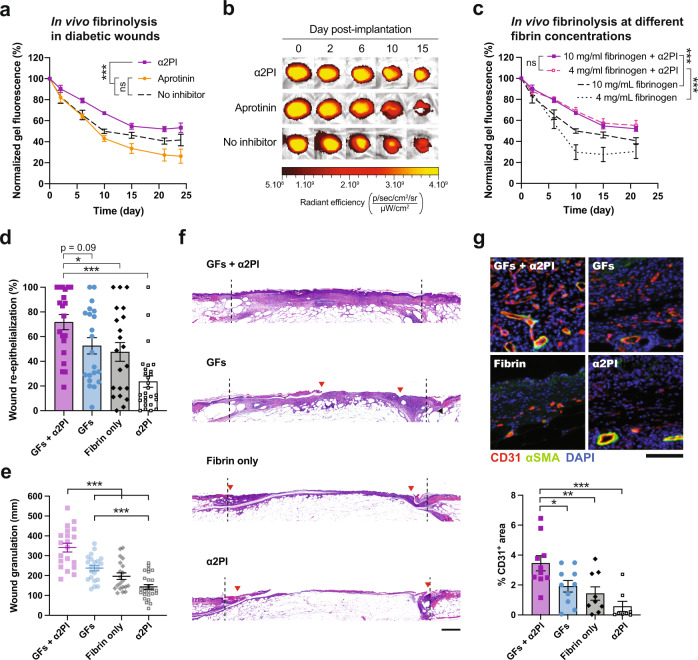
α2PI enhances fibrin-mediated delivery of engineered GFs and improves diabetic wound healing. In the wound healing experiments, 6 mm diameter wounds were surgically induced on the skin of the back of db/db mice and topically treated with fibrin gels once at day 0. As a GF treatment, 200 ng of VEGF-A-PlGF-2_123–144_ + 200 ng of PDGF-BB-PlGF-2_123–144_ were incorporated in the fibrin gels. **a** In vivo degradation of fibrin gels containing 15 μM of α2PI, aprotinin or no inhibitor upon topical application on diabetic wounds. Fibrin degradation was monitored via reduction in gel fluorescence measured by IVIS imaging (*n* = 6 gels/group). **b** Representative images of fibrin degradation on diabetic wounds, associated to **a**. **c** In vivo fibrinolysis with or without α2PI for fibrin gels made of 4 mg/mL or 10 mg/mL fibrinogen (*n* = 6 gels/group). **d**, **e** Quantification of diabetic wound closure **d** and granulation tissue **e** at day 10 upon topical application of fibrin gels containing engineered GFs and/or 3 μM of α2PI (n ≥ 20 wounds/group, pooled data from 2 independent experimental repeats). **f** Histological images of the wounds observed at day 10 upon different treatments with fibrin gels ± engineered GFs ± 3 μM α2PI. Images are representative of the wound granulation observed in the different groups (black dashed line = initial wound opening; red arrow = tip of the wound epithelial tongues; scale bar = 500 μm). **g** Representative images and quantification of angiogenesis in the center of the wounds at day 10 post-wounding. Quantification of wound angiogenesis was determined by the area of CD31^+^ endothelial cells over the area of granulation tissue analyzed by immunohistochemistry (*n* ≥ 8 wounds/group; scale bar = 100 μm). Mean ± SEM. Statistical comparisons were done using ANOVA test with Dunnett’s post-hoc test in **a**, **c**, **e**, **g**, and using Kruskal-Wallis test with Dunn’s post-hoc test in **d**: *p-value < 0.05, **p-value < 0.01, ***p-value < 0.001, ns non-significant.

We first examined the difference in fibrin gel degradation upon topical application on diabetic wounds when the gels were laden with the different protease inhibitors. We compared fibrin gels made of 10 mg/mL fibrinogen and 15 μM of α2PI, aprotinin or no inhibitor. Similar to the results in the subcutaneous implantation model, we observed that α2PI outperformed aprotinin in stabilizing fibrin on diabetic wounds, with more than 50% of the initial gel volume remaining present after 15 days (Fig. 3a, b). Surprisingly, aprotinin-containing gels seemed to degrade slightly faster than the ones without inhibitor, although this difference was not statistically significant. In this model, almost all gels stopped degrading after about 15 days, likely due to the completion of wound re-epithelialization at this time, which would reduce exposure to wound proteases.

Furthermore, we confirmed that gels made of 4 mg/mL of fibrin degraded at similar speed as those of 10 mg/mL when ladened with α2PI, in contrast to gels with no inhibitor that degraded significantly faster (Fig. 3c). This result, expected based on the ones obtained in the subcutaneous model, highlighted that α2PI permits adequate protection of low-concentration fibrin biomaterials for topical application of fibrin on wounds as well.

### Fibrin protection by α2PI improves GFs delivery in diabetic wound healing

By slowing fibrinolysis on wounds, we hypothesized that α2PI could enhance the delivery of GFs from fibrin gels, since controlled sustained release of GFs is known to enhance tissue healing. In this context, we had previously developed engineered variants of VEGF-A and PDGF-BB fused to a domain of placental-derived growth factor (PlGF)−2 to confer the GFs super-affinity to the extracellular matrix^28,29^. Particularly, we demonstrated that the engineered VEGF-A-PlGF-2_123–144_ and PDGF-BB-PlGF-2_123–144_ exhibit strong binding to fibrin matrices and mostly release upon fibrin proteolytic degradation, rather than by burst release from the matrix. Consequently, the presence of the engineered GFs was extended in wounds, leading to a significant improvement in tissue healing as compared to the wild-type GFs^19^.

Here, we questioned whether α2PI can further control the release of these engineered GFs by slowing fibrinolysis. We particularly analyzed in vitro the effect of α2PI on the release of VEGF-A-PlGF-2_123–144_ from fibrin gels (10 mg/mL of fibrinogen) upon exposure to plasmin (Fig. S5), considering that VEGF-A-PlGF-2_123–144_ and PDGF-BB-PlGF-2_123–144_ are expected to behave similarly^19^. We found that, in the absence of α2PI, VEGF-A-PlGF-2_123–144_ released over about 8 days from the fibrin gels, consistent with our previously published results^19^. Adding α2PI in the fibrin formulation impressively prolonged VEGF-A-PlGF-2_123–144_ release to over 16 days, which corresponds to the longevity of the fibrin gels in presence of plasmin (Figs. 1c and S5).

We then compared the healing of diabetic wounds treated with a combination of VEGF-A-PlGF-2_123–144_ and PDGF-BB-PlGF-2_123–144_ delivered in fibrin with or without α2PI. Specifically, we treated the wounds topically once, immediately after the wounding surgery, using low-concentration 4 mg/mL fibrin matrices containing 200 ng of each GF and 3 μM of α2PI. Tissue regeneration was evaluated 10 days later by histomorphometric analysis, quantifying wound re-epithelialization and granulation tissue formation, both being important parameters to monitor enhancement of wound healing. We found that re-epithelialization of wounds treated with GFs + α2PI was significantly better that those treated with fibrin, whereas GF only was not. However, the comparison between GFs+α2PI and GFs did not reach statistical significance (*p*-value = 0.09, Kruskal-Wallis test with Dunn’s post-hoc test) due to the high intra-group variability. Overall, treatment with GFs + α2PI, GFs only and fibrin respectively led to average wound closures of 72 ± 27%, 52 ± 30% and 47 ± 34% (mean ± standard deviation; Fig. 3d). Here, the minimal effect of GFs as compared to fibrin alone could be explained by the suboptimal degradation of the low-concentration fibrin gels that failed to sustain their presence in the wound bed. Surprisingly, treatment of wound with α2PI without GFs seemed to delay wound re-epithelialization, leading to a limited closure of 24 ± 24%. This highlights that the healing associated to the α2PI + GFs treatment was not caused by a direct therapeutic effect of the α2PI molecule per se, rather by prolonging exposure to the GF therapeutic.

Similar results were observed on wound granulation tissue formation, with the highest amount of granulation tissue found upon treatment with GFs delivered in α2PI-containing fibrin (Fig. 3e, f). In absence of α2PI, the GFs induced about 29% less granulation tissue, and fibrin alone about 43% less. Again, α2PI in fibrin in absence of GFs impaired wound healing as compared to fibrin alone. However, this apparent negative effect of α2PI alone should be carefully considered, since improved granulation tissue formation and trends toward improved re-epithelialization was observed upon treatment with α2PI at a higher concentration of 10 mg/mL fibrin (Fig. S6).

Because VEGF-A-PlGF-2_123–144_ and PDGF-BB-PlGF-2_123–144_ primarily enhance blood vessel formation and maturation in the wound bed, we analyzed wound angiogenesis by immunofluorescence, detecting CD31^+^ endothelial cells and αSMA^+^ pericytes and smooth muscle cells. We observed that wound angiogenesis was significantly improved in the center of the wounds when the GFs were delivered using fibrin supplemented with α2PI, as reflected by a higher percentage of the area covered by CD31^+^ endothelial cells (Figs. 3g and S7a). In addition, microscopic images revealed that the endothelial cells were surrounded by αSMA^+^ cells in these wounds, suggesting a stabilized and functional blood vessel structure. Similarly, treatment by GFs + α2PI enhanced angiogenesis as compared to GFs only at the wound edges (Fig. S7b).

Finally, we explored the performance of α2PI in enhancing the delivery of GFs from fibrin in the excessive presence of plasmin, considering that diabetic patients generally have more wound exudate than mice, which contains elevated plasmin levels^30^. To mimic this, we repeated the wound healing experiment as previously described yet incorporating 50 nM plasmin during fibrin gel preparation. Under these conditions, we found that GFs delivered with α2PI-supplemented fibrin also showed trends toward accelerated wound closure and significant increase in granulation tissue formation as compared to GFs only (Fig. S8).

### Crosslinking of α2PI into endogenous fibrin

In the clinic, one key application of aprotinin is as a hemostatic agent during coronary artery bypass graft surgery (CABG), to reduce bleeding loss and subsequent need for patient blood transfusion^12,14,17^. In this application, aprotinin is injected intravenously to the patient to stabilize the formation of endogenous fibrin clots, overall favoring sustained blood coagulation at anastomotic sites. Considering both the high efficacy of α2PI as compared to aprotinin in protecting fibrin materials, and its human origin, which confers a strong advantage to reduce adverse immune effects, we here aimed to evaluate α2PI for use as a potential hemostatic agent for systemic intravenous administration.

**Fig. 4:**
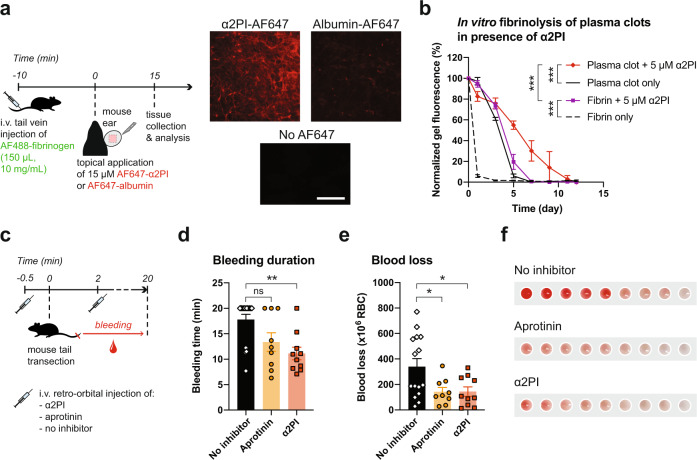
α2PI protects endogenous fibrin and reduces bleeding in a tail vein transection model in mice upon intravenous delivery. **a** Scheme of the experimental procedure and in situ crosslinking of α2PI in endogenous fibrin on wounds, as compared to non-binding albumin. Fluorescently-labelled α2PI or albumin was added directly upon wound induction on mice ear flap. The wounds were then extensively washed to remove unbound proteins, before microscopic imaging (representative images of *n* = 3, scale bar = 100 μm). **b** In vitro fibrinolysis of clots polymerized from unpurified mouse plasma (i.e. plasma clot) supplemented or not with 5 μM of α2PI and in the presence of 2.5 nM of plasmin. The plasma was mixed with 10% fluorescently-labelled exogenous fibrinogen prior to clotting to allow quantification of clot degradation via IVIS imaging. Mean ± SD. **c** In vivo tail bleeding model C57BL6/J in mice (*n* ≥ 9 mice/group). α2PI or aprotinin (180 μM, 100 μL) was injected retro-orbitally, 30 sec prior to full transection of the tail tip and 2 min after. The bleeding time was measured from the time of tail transection and up to 20 min later, during which the blood was collected for further quantification. **d** Bleeding time of mice injected with α2PI, aprotinin or no inhibitor. Statistical comparisons were done using Kruskal–Wallis test with Dunn’s post-hoc test. **e** Blood loss is quantified via the amount of lost red blood cells (RBC) upon injection of the different inhibitors. **f** Representative images of blood samples collected from mice treated with α2PI, aprotinin or no inhibitor (red intensity = increase in blood loss). Mean ± SEM if not stated otherwise. Statistical comparisons were done using ANOVA test with Dunnett’s post-hoc test if not stated otherwise: *p-value < 0.05, **p-value < 0.01, ***p-value < 0.001, ns non-significant.

We first confirmed that α2PI can effectively crosslink into endogenous fibrin. To do so, we topically applied fluorescently-labeled α2PI or albumin (used as a non-binding protein negative control) on wounded ears in mice for 15 min, immediately after wounding, after which we extensively washed the unbound proteins. Microscopy images revealed that α2PI but not albumin can clearly be detected after washing, in a meshed pattern similar to the one of a fibrin network (Fig. 4a). A similar experiment in mice injected intravenously with fluorescently-labeled exogenous fibrinogen further supported the co-localization of α2PI and fibrin (Fig. S9), suggesting the crosslinking of the exogenous α2PI into fibrin during polymerization.

We then examined the efficacy of α2PI to prevent endogenous fibrinolysis in vitro. We incorporated 5 μM of α2PI into plasma clots prepared from unpurified mouse plasma, known to contain about 2 mg/mL of fibrinogen^31–34^. Of note, the physiological concentration of fibrinogen in human plasma is in a similar range 1.5–4.5 mg/mL^35^. The plasma clots were polymerized with 25 mM CaCl_2_, after addition of 10% w/w exogenous fluorescent fibrinogen to allow fibrinolysis quantification. The longevity of the plasma clots was compared to ones of purified fibrin gels made of fibrinogen concentration (2.5 mg/mL) and polymerized with thrombin, Factor XIIIa and CaCl_2_. Overall, the plasma clots resisted fibrinolysis better than fibrin gels, likely due to the presence of endogenous α2PI and other protease inhibitors in the unpurified plasma (Fig. 4b). The addition of 5 μM α2PI in plasma clots further stabilized, increasing their longevity from 7 to 12 days.

### Therapeutic use of α2PI as a hemostatic agent

Based on the strong protective effects of α2PI on endogenous fibrin in vitro, we lastly explored the use of α2PI as a hemostatic agent in vivo upon intravenous delivery. We established a tail bleeding model in C57BL/6 J mice to measure blood coagulation upon tail transection. C57BL/6 J mice have been reported to exhibit higher blood loss in the tail-tip bleeding model than other common strains of mice, such as Balb/c and 129S1/Sv mice^34^. In this model, we administered α2PI or the clinically used aprotinin via retro-orbital intravenous injections 30 s before tail transection and 2 min after. The bleeding time and bleeding volume were assessed for up to 20 min (Fig. 4c). We observed that α2PI was able to significantly reduce bleeding time and blood loss as effectively as the clinical drug aprotinin (Fig. 4d, e). The effects of α2PI on blood loss reduction can also be visualized via the lowered intensity of red coloring in the collected blood samples (Fig. 4f). Of note, while we decided to set the experimental endpoint at 20 min after tail transection, the actual bleeding time of mice in absence of inhibitor delivery can reach 90 min (Fig. S10a), which underrepresents the benefits of α2PI on bleeding time when compared to the control group without inhibitor. In contrast, the major fraction of blood loss occurs in the first 20 min of bleeding (Fig. S10b).

## Discussion

Aprotinin is currently used in the clinic as a broad-spectrum serine protease inhibitor to stabilize fibrin biomaterials and to reduce bleeding loss during surgery. Nevertheless, the bovine origin of aprotinin raised immune-related safety concerns, particularly upon drug re-exposure. Therefore, in this study, we evaluated the efficacy of α2PI as a possible human substitute to aprotinin.

While α2PI is naturally present in human blood and can be effectively purified from it^36,37^, potentially making blood donation a source for its use as a drug, human-derived sourcing is limited by blood availability, a risk of blood-borne pathogen transmission and some batch-to-batch variability^38,39^. For these reasons, recombinant expression of α2PI presents a strong advantage for its clinical translation. Using transient transfection, we showed that α2PI can be successfully expressed in mammalian cells at a relatively high yield of 20 mg/L of production after purification (Fig. 1b). Further development of stable cell lines would certainly increase this yield, allowing for large scale clinical manufacturing. Importantly, we also demonstrated that the recombinant α2PI has similar bioactivity in inhibiting plasmin as the plasma-purified α2PI (Fig. S1).

We then demonstrated in vitro and in vivo in mice that α2PI vastly outperformed aprotinin in protecting fibrin, in the 3 main clinical or pre-clinical applications of aprotinin, i.e., in fibrin sealants, as a protease inhibitor to enhance fibrin-mediated drug delivery in wound healing, and as a hemostatic agent during surgery.

First, we highlighted that α2PI allowed stabilization of subcutaneously implanted fibrin biomaterials, mimicking an application in fibrin sealants. In this model, α2PI-containing implants lasted for more than a month, while the ones supplemented with aprotinin degraded in about 2 weeks. This significant difference in efficacy between aprotinin and α2PI can be partly explained by the retention profile of the different inhibitors into fibrin. Indeed, aprotinin is a low-molecular weight peptide that can diffuse out of fibrin implants whereas α2PI naturally contains a transglutaminase substrate domain α2PI_1–8_ that allows its covalent crosslinking into fibrin during its polymerization (Fig. 1a, Fig. 4a). Indeed, in a previous study, our laboratory had engineered the fusion protein aprotinin-α2PI_1–8_ and showed that subcutaneous implants made of fibrin supplemented with aprotinin-α2PI_1–8_ were still visible at 24 days, in a similar model^11^. This would suggest that α2PI and aprotinin-α2PI_1–8_ have comparable biological activity but that the lack of retention of aprotinin into fibrin is a main limiting factor to its apparent efficacy. Interestingly, we did not observe any significant effect of aprotinin on the longevity of the fibrin gels, as compared to fibrin alone (i.e., without inhibitor). This absence of efficacy of aprotinin could be explained by the low concentration of aprotinin used as compared to the clinical one as well as by the low concentration of fibrinogen, which possibly accelerates aprotinin release by reducing physical entrapment. Indeed, current fibrin sealant products use a high fibrinogen concentration of 22.5–45 mg/mL, for example in TISSEEL™ (Baxter)^9^ and Evicel (Johnson & Johnson)^10^. At such high concentrations, the duration of fibrin sealing clots upon delivery in patients lasts for about 6 days in the absence of aprotinin (Evicel) and 14 days when supplemented with aprotinin (TISSEEL)™^8,40^. While α2PI performance should be further evaluated side-by-side in humans to provide relevant comparison to these products, the results of this pre-clinical study overall suggest a strong potential of α2PI as compared to aprotinin.

In addition to extending fibrin duration, a potent inhibitor of fibrinolysis would permit reduction in fibrinogen concentration and therefore increase fibrin’s cost-effectiveness. Consequently, we studied the efficacy of α2PI in low-concentration fibrin biomaterials and showed in vitro that the longevity of fibrin was similar for gels made with 4 mg/mL and 12 mg/mL, allowing a 3 times reduction in concentration (Fig. 2c). This finding was then confirmed in vivo both in a subcutaneous implantation model and in a fibrinolysis model on diabetic skin wounds, in which 4 mg/mL fibrin gels degraded at similar speed as the 10 mg/mL ones in the presence of α2PI (Figs. 2d, 3c). While α2PI allow for substantial fibrinogen dose reduction, the minimal concentration of fibrinogen in a product would also depend on the specific application; in the case of fibrin sealants, for example, the fibrinogen concentration additionally need to ensure good tissue sealing properties, in addition to extended duration.

In a second application, we explored the use of α2PI to improve the delivery of fibrin-mediated GFs in diabetic wound healing. Diabetic wounds are hard to heal and constitute a major health concern^41^. Due to the underlying pathology, diabetic wounds remain in a chronic inflammation stage over months, characterized by a high proteolytic environment^24–26^. While not yet used as a clinical therapy for diabetic wound healing, the delivery of GFs via fibrin has been extensively assessed in pre-clinical research^28^, and aprotinin has been used to stabilize fibrin in this context as well. Using the db/db mouse model of type II diabetes, we showed that α2PI was again more effective in preventing fibrinolysis than aprotinin (Fig. 3a, b). We then tailored the delivery of VEGF-A-PlGF-2_123–144_ and PDGF-BB-PlGF-2_123–144_ in diabetic wounds by using α2PI in low-concentration fibrin materials. VEGF-A and PDGF-BB are GFs involved in angiogenesis and pericyte/mesenchymal stem cells recruitment, which are known to accelerate wound healing^42,43^. Importantly, PDGF-BB is the active compound of the product Regranex^®^ (Smith & Nephew) currently used as a therapy for diabetic foot ulcers. Here, we showed that α2PI-containing fibrin significantly enhanced the delivery of VEGF-A-PlGF-2_123–144_ and PDGF-BB-PlGF-2_123–144_, as indicated by improved angiogenesis at the wound center, increased granulation tissue formation and accelerated wound closure (Fig. 3d–g). We showed that α2PI did not provide good therapeutic effects per se in the used experimental settings, but rather that its presence modified the kinetics of GF release from fibrin (Fig. S5). Moreover, it is expected that α2PI protects the GFs from proteolysis, in addition to protecting fibrin, as it is known for example that VEGF-A contains plasmin-sensitive sites that reduce its activity upon cleavage^44^.

Lastly, we compared the efficacy of α2PI as substitute for aprotinin for use as a hemostatic agent during surgery, such as in CABG surgery, for which the major cause of excessive bleeding is the increase in plasmin activity^45–47^. In a tail bleeding model in mice, we showed that intravenous injection of α2PI has comparable hemostatic efficacy as aprotinin, both reducing bleeding time and blood loss as compared to untreated mice (Fig. 4d–f).

Therefore, α2PI appears as a highly competitive drug substitute to aprotinin in the main applications in which aprotinin is currently used. Importantly, the human origin of α2PI constitutes a major advantage, solving the immunological-based safety concerns associated to the bovine origin of aprotinin. While dose and safety studies of α2PI in large animal models and in human would be necessary for further clinical translation, it is expected that local and topical use of α2PI would be safe considering that a low dose seems sufficient to effectively protect exogenous fibrin biomaterials and that α2PI is the endogenous inhibitor of fibrinolysis in human. In contrast, more attention would be needed for its use as an intravenous agent, since a high blood level of α2PI has been associated with risks of ischemic stroke in patients^48^. Nevertheless, the use of α2PI in the context of surgery would be acute and designed to counteract the excessive activity of plasmin, in contrast to the chronic elevation of α2PI that was detected in patients with ischemic stroke. Particularly, the dose and blood half-life of α2PI upon intravenous delivery will likely be important factors to establish a safe therapeutic window.

Lastly, the translatability of α2PI as a clinical drug would also depend on its final production cost as a recombinant protein, which might be more expensive than the production of aprotinin from bovine tissue or as a synthetic peptide. Nevertheless, the crosslinking ability of α2PI and the subsequent low dose required for good efficacy, along with a possible reduction of fibrin dose and related expenses (particularly for local use in biomaterials), might counterbalance the production cost difference with aprotinin. Besides aprotinin, some small molecules inhibitors, such as tranexamic acid (TXA) and aminocaproic acid, have also been used as antifibrinolytic agents in the clinic. These are relatively inexpensive simple lysine analogs and have different mechanisms of action than the more complex aprotinin^47,49^. The advantages of TXA versus aprotinin remains controversial and depend on specific applications (e.g., type of cardiovascular surgery)^50^. However, many studies showed that aprotinin remains more effective that lysine analogs in fibrinolysis inhibition^51,52^. It would be important to evaluate side-by-side the efficacy and safety of the α2PI as compared to the use of TXA and aprotinin in specific applications.

Altogether, the clinical development of α2PI as a human-derived fibrinolytic inhibitor might be valuable in multiple applications of regenerative medicine.

## Methods

### Cloning, expression, and purification of α2PI

Human α2PI DNA sequence was purchased from GenScript and cloned into pXLG plasmid under a CMV promoter and using an IgGκ signal peptide, for expression in mammalian cells. A 8x histidine tag was added at the N-terminus of the α2PI sequence. Suspension-adapted Human Embryonic Kidney (HEK) 293-F cells were transfected with the α2PI plasmid DNA using polyethyleneimine. Cell culture supernatant was collected 7 days after the transfection and was purified by affinity-mediated purification using HisTrap HP his-tag purification column (GE Healthcare). The column was equilibrated with 20 mM NaH_2_PO_4_, 0.5 M NaCl, pH 7.4. The cell culture supernatant was then loaded through column, and the column was washed with the equilibration buffer. The protein was then eluted with 20 mM NaH_2_PO_4_, 0.5 M NaCl, 1 M imidazole, pH 8. Fractions of the elution were collected, dialyzed against Tris-Buffered Saline (TBS: 150 mM NaCl, 20 mM Tris, pH 7.4), sterile-filtered at 0.22 μm and stored at −80 °C until use. Purified proteins were visualized on SDS-PAGE gels and identified by western blot analysis using anti-his antibody (Abcam, ab1187, dilution 1:10,000) and anti-α2PI antibody (Abcam, ab62770, dilution 5 μg/mL). Uncropped SDS-PAGE gel and western blots can be found in Fig. S11.

### Fluorescent fibrinogen preparation

Lyophilized human fibrinogen (FIB3, pg, vWF & Fn depleted, Enzyme Research Laboratories) was dissolved at 37 °C in HEPES buffer (150 mM NaCl, 20 mM HEPES, pH 7.6) and extensively dialyzed. The fibrinogen was then concentrated using Amicon Ultra Centrifugal Filters (Millipore) and further stored at −80 °C until use. Fluorescent fibrinogen was prepared by mixing 0.1 mg Alexa Fluor-680 or Alexa Fluor-488 NHS ester (Invitrogen) with 100 mg of fibrinogen in 0.1 M sodium bicarbonate buffer. The reaction was incubated for 2 h at room temperature under continuous shaking. The fluorescent fibrinogen was desalted using Zeba Spin Desalting Columns (Thermo Scientific) to remove unconjugated free dyes. The fluorescent fibrinogen was quantified by nanodrop and stored at −80 °C.

### Proteolytic inhibition of fibrinolysis in vitro

Fibrin gels (70 μL, *n* = 3 per group) were made of 10 mg/mL fibrinogen containing 25% w/w fluorescent fibrinogen, 2 U/mL thrombin, 4 U/mL Factor XIIIa (pre-incubated with thrombin), and 5 mM CaCl_2_ in HEPES buffer, in which 1 μM inhibitors α2PI, aprotinin (Roche), and KPI-α2PI_1–8_ (in-house production)^13^ were added. KPI-α2PI_1–8_ was produced as described in the section “Cloning, expression, and purification of α2PI”. The gels were incubated for 1 h at 37 °C with 5% CO_2_ to ensure complete polymerization, and then transferred into a 24-well cell culture plates and incubated in 1 ml release buffer (Tris 20 mM, NaCl 150 mM, 0.1% BSA, Pen/Strep, pH 7.4) containing 2.5 nM plasmin (Roche). The plate was kept at 37 °C with 5% CO_2_ until the gels were fully degraded. The plasmin-containing buffer was daily refreshed. The gel volume were quantified over time by IVIS Spectrum system (Perkin-Elmer) via fluorescence measurements and analyzed using the recommended software (PerkinElmer). The percentages of remaining gel was normalized to the total radiant efficiency at day 0. The same method was used for the in vitro fibrinolysis assessment of different inhibitor concentrations (0.1 μM, 0.32 μM, 1 μM, 3.2 μM, 10 μM, and 15 μM), different fibrinogen concentrations (4 mg/mL, 6 mg/mL, 8 mg/mL, 10 mg/mL, and 12 mg/mL), and at different plasmin concentrations (2.5 nM or 25 nM). Fibrin gels of 100 μL were used for the bioactivity comparison of recombinant versus plasma-purified α2PI. Gels were considered degraded when ≤1% of their initial fluorescence was remaining. For the experiment using non-fluorescent fibrin gels, gels were prepare using the same compositions except that 10 mg/mL of non-fluorescent fibrin was used. The gels were imaged over time using a gel imaging system (Bio-Rad ChemDoc XRS) and gel diameter was measured and normalized to the diameter at day 0.

### Proteolytic inhibition of fibrinolysis in vivo in a subcutaneous implantation model

All in vivo experimentation was approved by the IACUC of University of Chicago. Fibrin gels (100 μL) were prepared as detailed before using 15 μM of the inhibitors α2PI, aprotinin or KPI-α2PI_1–8_. Female BALB/c mice at 8–10 weeks age were anesthetized by isoflurane inhalation and placed on a heating pad. Buprenorphine was injected subcutaneously at 0.1 mg/kg as analgesia, and artificial tears ointment was applied to the eyes of mice. The back of the mice was shaved, and disinfected with betadine wipes, followed by 70% ethanol wipes. Two incisions of about 8 mm were created on the skin, one on each side of the spine, and subcutaneous pockets were created using sterile scissors and forceps. The fibrin gels (*n* = 5 gels/group) were implanted subcutaneously in the back of the mice, for a total of 2 gels of different conditions per mouse. Prolene 4-0 sutures (Ethicon) were used to close the incisions. The degradation of the fibrin gels was quantified by IVIS Spectrum system via fluorescence detection and analyzed as described above in the “Proteolytic inhibition of fibrinolysis” in vitro section.

### Plasmin-mediated release of VEGF-A-PlGF-2_123–144_ from fibrin

Fibrin gels (70 μL; *n* = 4 gels per group) were made of 10 mg/mL fibrinogen, 2 U/mL thrombin, 4 U/mL Factor XIIIa, and 5 mM CaCl_2_ in HEPES buffer, in which 1 μM of α2PI and 200 ng VEGF-A-PlGF-2_123–144_ and PDGF-BB-PlGF-2_123–144_ were added, were incubated for 1 h at 37 °C with 5% CO_2_. Then, the fibrin gels were transferred into a 24-well cell culture plates and incubated in 1 ml release buffer (Tris 20 mM, NaCl 150 mM, 0.1% BSA, Pen/Strep, pH 7.4) containing 2.5 nM plasmin (Roche, #10602361001). The plate was kept at 37 °C with 5% CO2 until the gels were fully degraded. The plasmin-containing buffer was daily replaced, collected and stored at −20 °C. The daily release of VEGF-A was quantified by ELISA (Human VEGF DuoSet ELISA, R&D systems) as instructed by the manufacturer and normalized to the total released amount.

### Proteolytic inhibition of fibrinolysis in vivo on diabetic wounds in db/db mice

All in vivo experimentation was approved by the IACUC of University of Chicago. Male db/db mice of 10–12 weeks old were injected with buprenorphine at 0.1 mg/kg subcutaneously as analgesia. The back of mice was shaved and disinfected with betadine wipes followed by 70% isopropyl alcohol wipes. After disinfection, 4 wounds were created on the back of mice using a 6-mm biopsy punch with two wounds on each side of the spine. After wounding, fibrin gels (70 μL) were made by mixing 10 mg/mL or 4 mg/mL fibrinogen of 25% w/w fluorescent fibrinogen, 2 U/mL thrombin, 4 U/mL Factor XIIIa, 5 mM CaCl_2_ in HEPES buffer and α2PI or aprotinin inhibitors (15 μM or 3 μM), and directly polymerized on wounds (*n* = 6 gels/group). The different inhibitor treatments were randomized on the wounds so that each mouse received gels from different treatment groups. The wounds were covered by hydrofilms (Hartmann). The degradation of the fibrin gels on wounds were quantified by IVIS Spectrum system via fluorescence detection as described above in the “Proteolytic inhibition of fibrinolysis” in vitro section. Mice were anesthetized by isoflurane inhalation during imaging.

### Quantification of plasminogen content in wounds of db/db mice

Four male db/db mice at 10–12 weeks age were included for the ELISA analysis. Animal experimentation was approved by the IACUC of University of Chicago. Isoflurane was applied to the mice by inhalation as anesthetization, with 4% at the beginning, and 2% for maintenance. The mice were placed on a heating pad. Buprenorphine was injected subcutaneously at dosage of 0.1 mg/kg as analgesia, and artificial tear ointment was applied to the eyes. The back of the mice was shaved, and disinfected with betadine wipes, followed by 70% ethanol wipes. Four wounds were created using 6 mm punch biopsy on the back of each mouse, with two wounds on each side of the spine. The back of the mice are covered by hydrofilm (Hartmann), with the edges of the hydrofilms sealed using 1× Histoacryl BLUE glue (B. Braun Surgical). At day 3 or day 7 after the surgery, the mice were euthanized. The wounds of the mice were extracted using 8 mm punch biopsy, immediately frozen in liquid nitrogen and transferred to −80 °C for storage until further analyses. ELISA and BCA assays were performed to analyze content of plasminogen in wounds. The wounds were homogenized using lysis buffer (Tris-HCl 50 mM, NaCl 120 mM, EDTA 1 mM, EGTA, 1% NP-40, dithiothreitol 1 mM). ELISA was performed on the wounds lysate using Plasminogen Total Mouse ELISA kit (Abcam), and BCA assay was performed using Pierce BCA Protein Assay Kit (Thermo Scientific). The contents of plasminogen in wounds were calculated in term of ng plasminogen/mg total protein.

### Skin wound healing of db/db mice using fibrin gel

The wounding procedure on db/db male mice was the same as described before. After wounding, fibrin gels (50 μL) were polymerized directly on the wounds by mixing 4 mg/mL fibrinogen, 2 U/ml thrombin, 4 U/mL Factor XIIIA, 5 mM CaCl_2_ in HEPES buffer with α2PI (3 μM), VEGF-A-PlGF-2_123–144_ (200 ng/wound), and PDGF-BB-PlGF-2_123–144_ (200 ng/wound) (*n* ≥ 20 wounds/group). The different treatments were randomized on the wounds so that each mouse received gels from different treatment groups. After polymerization of fibrin gel, nylon splints (6 mm or 8 mm internal diameter) were sticked to the surrounding of the wound using 1x Histoacryl BLUE glue (B. Braun Surgical). The top of the splints was covered by hydrofilms (Hartmann), with a few holes created by needle enabling ventilation. Mice were euthanised after 10 days and wounds were collected and fixed using 4% paraformaldehyde (PFA) in PBS overnight at 4 °C. The fixed wounds were embedded in paraffin and cut into 5 μm thick cross-sections. The slides were stained using H&E staining. Images were taken using microscope (Leica DMi8) to analyze wound closure and granulation tissue formation using Fiji software (ImageJ).

### Preparation of fibrin gel containing plasmin

The wound healing model is the same than the one described before, except that the wounds were directly covered with hydrofilms, without splints. Plasmin (50 nM) was added together with 4 mg/mL fibrinogen, 2 U/ml thrombin, 4 U/mL Factor XIIIA, 5 mM CaCl_2_ in HEPES buffer with α2PI (3 μM), and then mixed with VEGF-A-PlGF-2_123–144_ (200 ng/wound) and PDGF-BB-PlGF-2_123–144_ (200 ng/wound) on the wounds.

### Histology analysis for wound closure and granulation

The mice were euthanized 10 days after the surgery, with the wounded tissues dissected and fixed using 4% PFA in PBS overnight at 4 °C. The fixed wounds were embedded into paraffin and sectioned at 5 μm to obtained cross-sections at the center of the wounds. The slides were stained using hematoxylin and eosin (H&E) staining. Images were taken using microscope (Leica DMi8) and wound closure and granulation tissue formation in wounds were analyzed using Fiji (ImageJ Open Source Software). The distance between the wound edges and between the regenerated epithelium edges were measured to calculate the percentage of wound closure. The area of granulation tissue was measured and normalized to the initial wound size (detected by the edges of the panniculus carnosus subcutaneous muscle).

### Immunohistochemistry staining on wounds and quantification

Paraffin sections of the wounds (*n* ≥ 8 wounds/group) were dewaxed and rehydrated using serial washes in xylene, ethanol (100%, 96%, and 70%), and water. The antigens of the tissues were retrieved in citrate buffer (10 mM sodium citrate, 0.05% Tween 20, pH 6.0). After antigen retrieval, the slides were washed using TBS (150 mM NaCl, 20 mM Tris, pH 7.4). The tissues on the slides were blocked with 5% casein in PBS (blocking buffer) for 2 h at room temperature, and then stained with anti-CD31 (Abcam, ab28364, dilution 1:50) and anti-alpha smooth muscle actin (αSMA; Sigma-Aldrich, A2547, dilution 1:200) in the blocking buffer overnight at 4 °C. After washes in TBS, secondary antibodies and DAPI were incubated for 2 h at room temperature. Finally, the slides were washed again and mounted for fluorescent microscopy imaging using a Leica DMi8 microscope. Angiogenesis was quantified using Fiji (ImageJ) by thresholding CD31^+^ area and dividing by the area of the granulation tissue in the regions of interest (wound center or wound edges).

### Fluorescent conjugation of α2PI and albumin

Fluorescent α2PI and albumin were prepared by mixing Alexa Fluor-647 NHS ester (Invitrogen) with α2PI or albumin at 10:1 molar ratio in 0.1 M sodium bicarbonate buffer for 2 h at room temperature with continuous shaking. The fluorescent labeled protein was purified using Zeba Spin Desalting Columns (Thermo Scientific) to remove unconjugated free dyes, and then stored at −80 °C until use.

### Binding of α2PI to fibrin on mice ear wounds

All in vivo experimentation was approved by the IACUC of University of Chicago. Balb/C mice of 8–15 weeks old were anesthetized by inhalation (*n* = 3 mice/group). Alexa Fluor-488-labelled fibrinogen (150 μL of 10 mg/mL) was intravenously injected to each mice prior to the surgery. Ten minutes later, the top skin and cartilage of the mice ears were delicately removed using a surgical blade, and 20 μL of the Alexa Fluore-647-labeled α2PI or albumin (15 μM) was pipetted on the ear and incubated for 15 minutes under coverslips to prevent drying. The mice were then directly euthanized and the ears were dissected and washed with 50 mL TBS buffer for 2 h at 4 °C. The wash process was repeated twice to remove unbound fluorescent proteins. After the washes, the mice ears were fixed overnight using a zinc fixative buffer, washed with TBS, cleared and mounted with Benzyl Alcohol/Benzyl Benzoate (BABB) before imaging.

### Proteolytic fibrinolysis of plasma gel in vitro

Immediately after euthanasia of C57BL/6 J mice, blood was withdrawn by cardiac puncture. The whole blood was mixed with sodium citrate (100 mM, pH 7.4–7.8) at 9:1 ratio to prevent coagulation, and centrifuged at 1500 × *g* for 10 min. The plasma supernatant was collected and froze at −80 °C until use. Fluorescent fibrinogen (0.2 mg/mL final, about 10% w/w) was mixed to mouse plasma to enable visualization of gel degradation by fluorescent imaging. Recombinant α2PI (5 μM) was added in the fibrinogen mix. To induce clotting, CaCl_2_ in HEPES buffer was added to the fluorescent plasma mix to a 25 mM final concentration. Plasma clots were incubated for 1 h at 37 °C with 5% CO_2_ to ensure complete polymerization. The plasma gels were then transferred into a 24-well cell culture plate, 1 gel per well, and incubated in 1 ml release buffer (Tris 20 mM, NaCl 150 mM, 0.1% BSA, Pen/Strep, pH 7.4) containing 25 nM plasmin (Roche). The plate was kept at 37 °C with 5% CO_2_ until all the gels were fully degraded. The plasmin-containing buffer was daily refreshed, and the imaging was performed as previously described.

### Tail bleeding model in mice for in vivo coagulation test

Female C57BL/6 J mice (8–20 weeks, randomly distributed between the groups, *n* ≥ 9 mice/group) were anesthetized by isoflurane inhalation (5% induction and 3.5% maintenance). The animal was placed on a 32 °C heating plate during the surgery to maintain body temperature. Protease inhibitors (100 μL at 178 μM) or HEPES buffer (150 mM NaCl, 20 mM HEPES, pH 7.6) was given to the mice by retro-orbital intravenous injection and, 30 seconds later, the mice tails were fully transected at 1 mm from tip of the tail using a razor blade guillotine that amputates the tip at a 90° angle. A new razor blade was used for each mouse. The tail was then submerged in 10 mL PBS at room temperature. Two minutes after the tail transection, a second dose of protease inhibitor (100 μL at 178 μM) or HEPES buffer was injected retro-orbitally in the other eye of the mice. Bleeding of the tail was visually observed for up to 20 min after the transection, if not stated otherwise. A bleeding time of 20 min was assigned to the mice that were not able to stop bleeding after 20 min. The collected blood was finally quantified by counting the total number of red blood cells using a hemocytometer. The total number of RBCs was used as the indication of blood volume loss. These experiments were repeated by 2 different experimenters.

### Statistical analysis

Results are presented as mean ± standard deviation (SD) for in vitro experiments and mean ±standard error of the mean (SEM) for in vivo experiments. Statistical analysis were performed using Prism 9 (GraphPad). Comparisons between two groups were done using Student’s t-test. Comparisons between 3 or more groups were done using ANOVA test with Dunnett’s post-hoc test, or using Kruskal-Wallis test with Dunn’s post-hoc test for wound re-epithelialization and for bleeding time. In fibrinolysis experiments, statistical differences were reported when the groups were found different at least one of the timepoint. Tests were two-sided and statistical significance was reported as follow: *p-value < 0.05, **p-value < 0.01, ***p-value < 0.001. Non-significant differences were labeled as “ns”.

### Reporting summary

Further information on research design is available in the Nature Research Reporting Summary linked to this article.

## Supplementary information

Liu et al Supplementary Figures

REPORTING SUMMARY

## Data Availability

All data are included in the manuscript and supplementary materials.
