# Supplementary material for: Therapeutic use of α2-antiplasmin as an antifibrinolytic and hemostatic agent in surgery and regenerative medicine
Source: NPJ Regen Med. 2022 Jun 30;7:34. doi: 10.1038/s41536-022-00230-x (PMC9246914; doi:10.1038/s41536-022-00230-x)
Supplement: Supplementary file 1 — Liu et al Supplementary Figures [file 41536_2022_230_MOESM1_ESM.pdf]

Supplementary Information

Therapeutic use of α2-antiplasmin as an antifibrinolytic and hemostatic agent in surgery and regenerative medicine

Jialu Liu[1], Ani Solanki[2], Michael J. V. White[1], Jeffrey A. Hubbell[1, 3, 4]*, Priscilla S. Briquez[1, *]

Author Affiliations:

[1] Pritzker School of Molecular Engineering, University of Chicago, Chicago 60637 IL, USA

[2] Animal Resources Center, University of Chicago, Chicago, IL 60637, USA

[3] Committee on Immunology, University of Chicago, Chicago IL 60637, USA

[4] Committee on Cancer Biology, University of Chicago, Chicago, IL 60637, USA

* Correspondence to: jhubbell@uchicago.edu, pbriquez@uchicago.edu

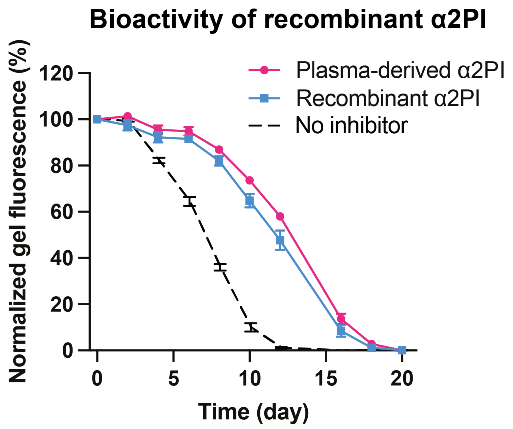

Supplementary Figure 1. **Activity of α2PI in preventing fibrinolysis.** Comparison of the bioactivity of recombinant α2PI as compared to the commercially available plasma-purified α2PI (1 μM). Recombinant α2PI has similar activity than the human plasma-derived one.

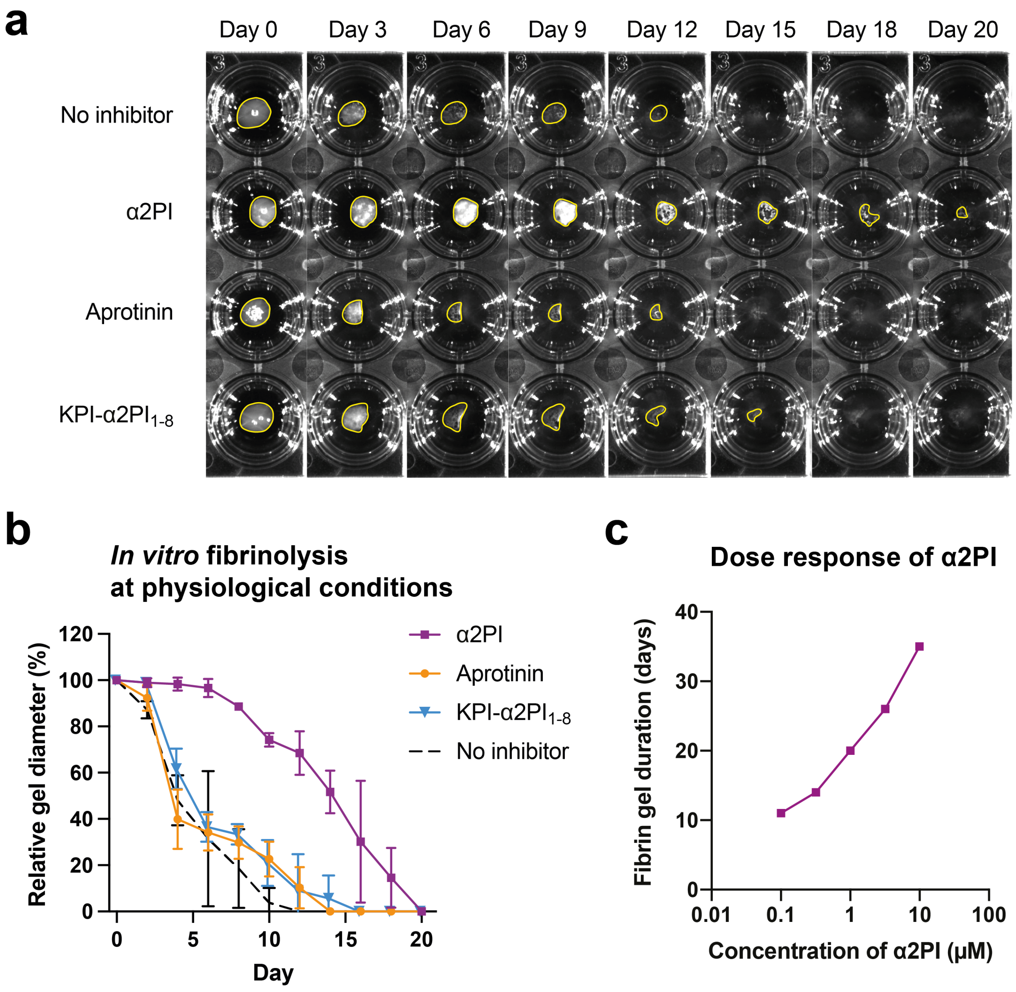

Supplementary Figure 2. **α2PI protects fibrin gels from plasmin-mediated degradation. (a)** Representative images of fibrin gel degradation (gels are circled in yellow) and **(b)** quantification of relative fibrin gel diameters over time, using the same conditions as in the experiment in Fig. 1c,d with non-fluorescent fibrin (n=3 gels/group). **(c)** Fibrin gel duration in function of the α2PI concentration in presence of 2.5 nM of plasmin, as presented in Fig. 1e. Degradation time is defined as the day at which >99% of the fibrin was degraded.

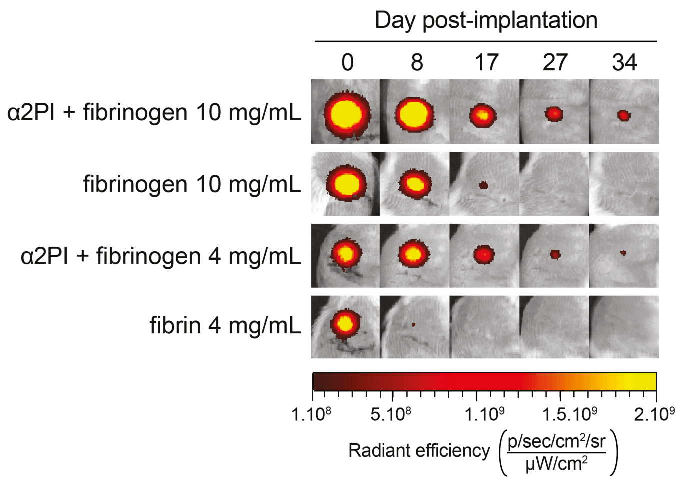

Supplementary Figure 3. **α2PI protects fibrin subcutaneous implants made of low-concentration fibrinogen.** Representative images of the degradation of fibrin gels, made of 10 mg/mL or 4 mg/mL fibrinogen, containing 15 μM of α2PI or no inhibitor when implanted subcutaneously, as presented in Fig. 2d.

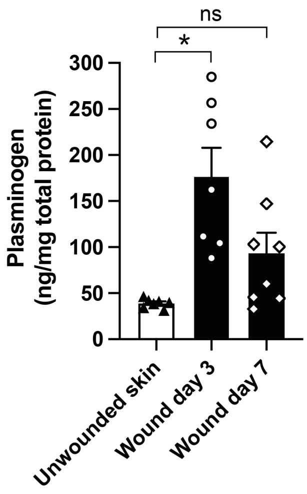

Supplementary Figure 4. **Plasminogen concentration in skin and wounds of db/db mice.** Full-thickness skin wounds were surgically performed on db/db mice at day 0. The wounds were harvested at day 3 and 7 post-wounding for analysis of plasminogen content and compared to the plasminogen level detected in unwounded skin in db/db mice (n≥7 wounds/group). Mean ± SEM. Statistical comparison was done using ANOVA test with Dunnett's post-hoc test: *p-value<0.05, **p-value<0.01, ***p-value<0.001, "ns": non-significant.

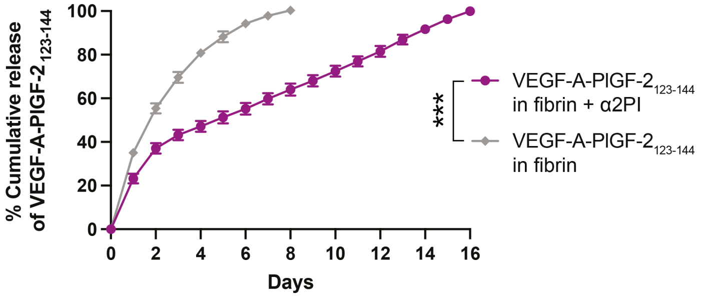

Supplementary Figure 5. **Gradual release of VEGF-A-PlGF-2$_{123\text{-}144}$ from fibrin gel supplemented with α2PI, in presence of plasmin.** Fibrin gels made of 10 mg/mL fibrinogen and 200 ng of VEGF-A-PlGF-2$_{123\text{-}144}$ were supplemented or not with 1 µM of α2PI, and incubated in 2.5 nM of plasmin. α2PI slowed the release of VEGF-A-PlGF-2$_{123\text{-}144}$. Mean ± SD. Statistical comparison was done using Student's t-test: *p-value < 0.05, **p-value < 0.01, ***p-value<0.001, "ns": non-significant.

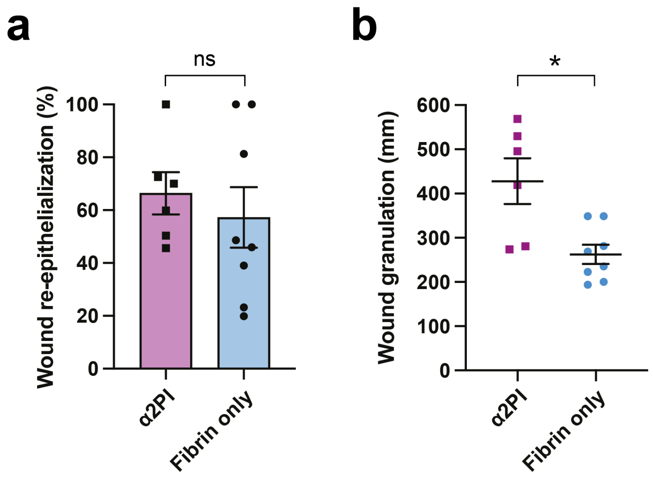

Supplementary Figure 6. **Diabetic wound healing upon topical treatment with fibrin gels made of 10 mg/mL fibrinogen, with or without 3 μM α2PI.** Histomorphometric analyses were performed at day 10 post-wounding. **(a)** Quantification of wound closure. **(b)** Quantification of wound granulation tissue formation. Mean ± SEM. Statistical comparisons were done using Mann-Whitney test in panel a and using Student's t-test in panel b: *p-value<0.05, **p-value<0.01, ***p-value<0.001, "ns": non-significant.

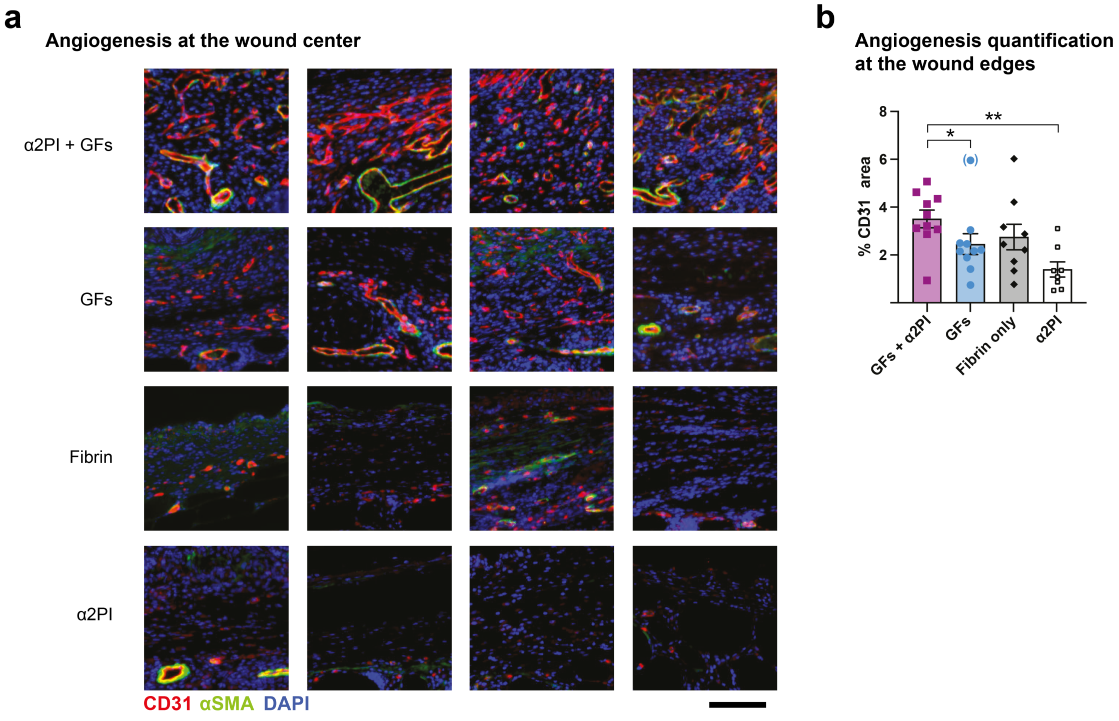

***Supplementary Figure 7.* Angiogenesis at the center and edges of the wounds at day 10 post-wounding. (a)** Representative images of angiogenesis at the wound center upon treatment with GFs delivered in fibrin supplemented or not with α2PI (4 different wounds per group are presented; scale bar = 100 μm). **(b)** Quantification of $CD31^+$ area over the area of granulation tissue at the edges of the wounds upon different treatments (n≥8 wounds/group). One outlier in the GFs group was excluded for statistical analysis (bracketed). $CD31^+$ is a marker for endothelial cells. Mean ± SEM. Statistical comparisons were done using ANOVA test with Dunnett's post-hoc test: *p-value<0.05, **p-value<0.01, ***p-value<0.001, "ns": non-significant.

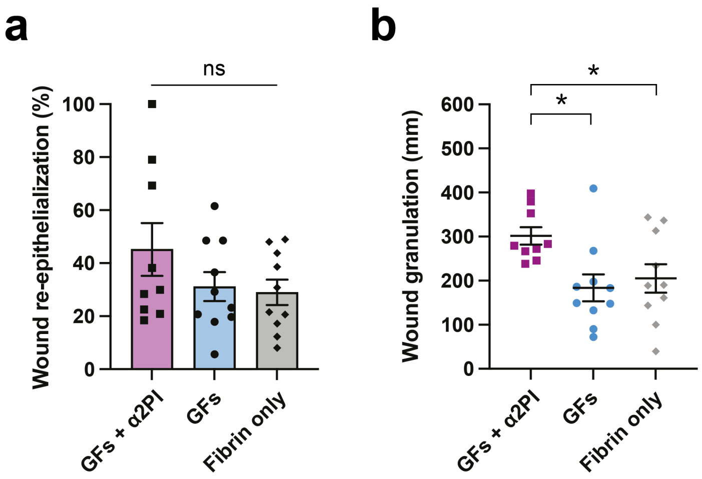

Supplementary Figure 8. **Diabetic wound healing upon topical treatment with fibrin gels containing engineered GFs with or without 3 μM α2PI in presence of high plasmin.** Fibrin gels containing different treatments were supplemented with 50 nM of exogenous plasmin, as a model to recapitulate the increased amount of highly proteolytic wound fluid present in human diabetic wounds. Wounds were analyzed by histomorphometry at day 10 post-wounding (n≥9 per group). **(a)** Quantification of wound closure. **(b)** Quantification of granulation tissue formation. Mean ± SEM. Statistical comparisons were done using Kruskal-Wallis test in panel a and ANOVA test with Dunnett's post-hoc test in panel b: *p-value<0.05, **p-value<0.01, ***p-value<0.001, "ns": non-significant.

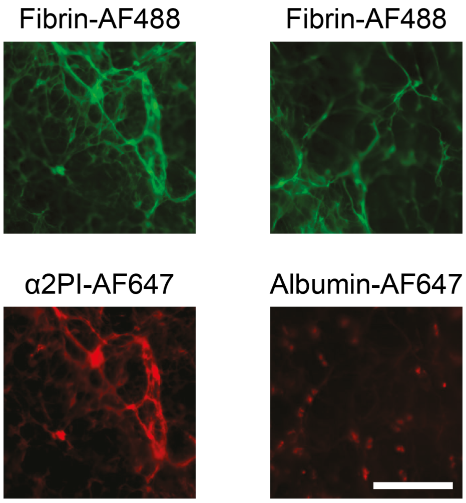

Supplementary Figure 9. **α2PI colocalizes with fibrin matrix.** Mice were intravenously injected with fluorescent fibrinogen before induction of ear flap wound. Fluorescent α2PI was directly applied on top of the wound. The ear wound was extensively washed prior to imaging. Scale bar = 50 μm.

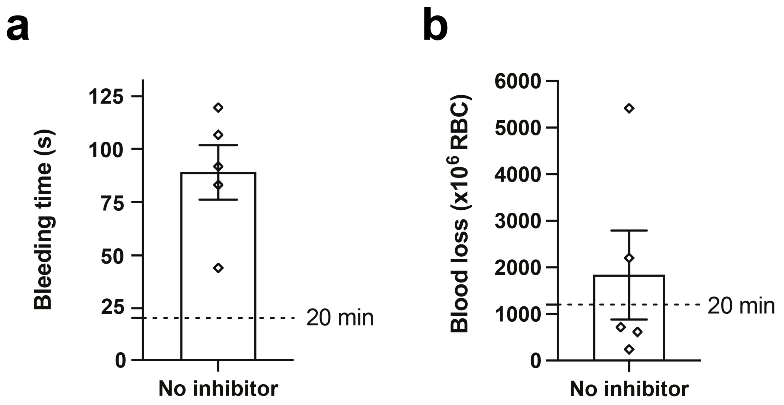

Supplementary Figure 10. **Bleeding model characterization.** The same model than used in Fig. 4 was used, but the endpoint was determined when the bleeding stopped, instead of at max. 20 min (n=5 mice). **(a)** Bleeding time when mice are not injected with any protease inhibitors. **(b)** Blood loss when mice are not injected with any protease inhibitors. Mean ± SEM.

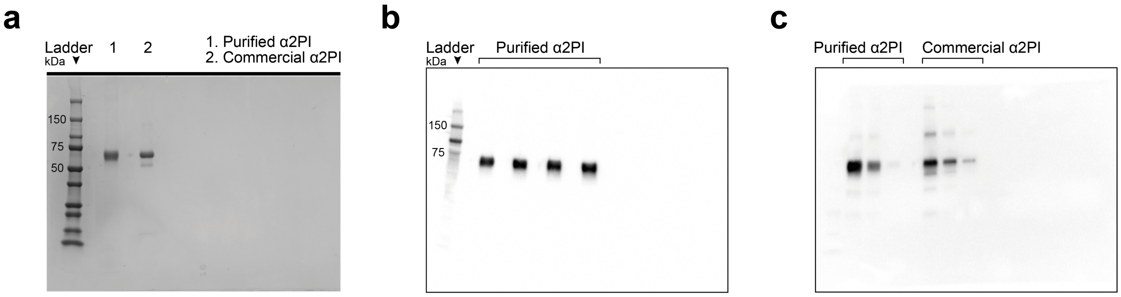

Supplementary figure 11. **Uncropped and unprocessed SDS-PAGE and western blots. (a)** Uncropped SDS-PAGE of purified and commercial α2PI. **(b)** Uncropped western blot of purified α2PI using anti-his antibody for the detection of α2PI. **(c)** Uncropped western blot of purified and commercial α2PI using anti-α2PI for the detection of α2PI.
