# Supplementary material for: Therapeutic use of α2-antiplasmin as an antifibrinolytic and hemostatic agent in surgery and regenerative medicine
Source: NPJ Regen Med. 2022 Jun 30;7:34. doi: 10.1038/s41536-022-00230-x (PMC9246914; doi:10.1038/s41536-022-00230-x)
Supplement: Supplementary file 2 — REPORTING SUMMARY [file 41536_2022_230_MOESM2_ESM.pdf]

nature portfolio

Corresponding author(s): Hubbell*, Briquez*

Last updated by author(s): Apr 8, 2022

Reporting Summary

Nature Portfolio wishes to improve the reproducibility of the work that we publish. This form provides structure for consistency and transparency in reporting. For further information on Nature Portfolio policies, see our Editorial Policies and the Editorial Policy Checklist.

Statistics

For all statistical analyses, confirm that the following items are present in the figure legend, table legend, main text, or Methods section.

n/a	Confirmed	
☐	☒	The exact sample size (n) for each experimental group/condition, given as a discrete number and unit of measurement
☐	☒	A statement on whether measurements were taken from distinct samples or whether the same sample was measured repeatedly
☐	☒	The statistical test(s) used AND whether they are one- or two-sided<br>*Only common tests should be described solely by name; describe more complex techniques in the Methods section.*
☒	☐	A description of all covariates tested
☐	☒	A description of any assumptions or corrections, such as tests of normality and adjustment for multiple comparisons
☐	☒	A full description of the statistical parameters including central tendency (e.g. means) or other basic estimates (e.g. regression coefficient) AND variation (e.g. standard deviation) or associated estimates of uncertainty (e.g. confidence intervals)
☒	☐	For null hypothesis testing, the test statistic (e.g. F, t, r) with confidence intervals, effect sizes, degrees of freedom and P value noted<br>*Give P values as exact values whenever suitable.*
☒	☐	For Bayesian analysis, information on the choice of priors and Markov chain Monte Carlo settings
☒	☐	For hierarchical and complex designs, identification of the appropriate level for tests and full reporting of outcomes
☒	☐	Estimates of effect sizes (e.g. Cohen's d, Pearson's r), indicating how they were calculated

Our web collection on statistics for biologists contains articles on many of the points above.

Software and code

Policy information about availability of computer code

Data collection	IVIS Spectrum system (Perkin-Elmer), BioRad ChemiDoc
Data analysis	Fiji (ImageJ Open Source Software), Prism 9 (GraphPad), IVIS Spectrum system (Perkin-Elmer), Microsoft Excel.

For manuscripts utilizing custom algorithms or software that are central to the research but not yet described in published literature, software must be made available to editors and reviewers. We strongly encourage code deposition in a community repository (e.g. GitHub). See the Nature Portfolio guidelines for submitting code & software for further information.

Data

Policy information about availability of data

All manuscripts must include a data availability statement. This statement should provide the following information, where applicable:

- Accession codes, unique identifiers, or web links for publicly available datasets
- A description of any restrictions on data availability
- For clinical datasets or third party data, please ensure that the statement adheres to our policy

All data are included in the manuscript and supplementary materials.

March 2021

Field-specific reporting

Please select the one below that is the best fit for your research. If you are not sure, read the appropriate sections before making your selection.

☒ Life sciences ☐ Behavioural & social sciences ☐ Ecological, evolutionary & environmental sciences

For a reference copy of the document with all sections, see nature.com/documents/nr-reporting-summary-flat.pdf

Life sciences study design

All studies must disclose on these points even when the disclosure is negative.

Sample size	Sample size were chosen prior to experimentation based on publications using same or very similar models.
Data exclusions	There is no data exclusion.
Replication	All experiments were repeated at least twice and results were consistently reproduced. Experiments were repeated with 2 different experimenters.
Randomization	In in vivo experiments, different conditions were randomized in each mouse.
Blinding	The experiments were not blinded but were reproduced by independent researchers.

Reporting for specific materials, systems and methods

We require information from authors about some types of materials, experimental systems and methods used in many studies. Here, indicate whether each material, system or method listed is relevant to your study. If you are not sure if a list item applies to your research, read the appropriate section before selecting a response.

Materials & experimental systems

n/a	Involved in the study	
☐	☒	Antibodies
☐	☒	Eukaryotic cell lines
☒	☐	Palaeontology and archaeology
☐	☒	Animals and other organisms
☒	☐	Human research participants
☒	☐	Clinical data
☒	☐	Dual use research of concern

Methods

n/a	Involved in the study	
☒	☐	ChIP-seq
☒	☐	Flow cytometry
☒	☐	MRI-based neuroimaging

Antibodies

Antibodies used	anti-CD31 (Abcam, ab28364); anti-alpha smooth muscle actin (αSMA; Sigma-Aldrich, A2547)
Validation	By the company primarily. We did confirm the absence of nonspecific staining by performing immunostaining controls using secondary antibody.

Eukaryotic cell lines

Policy information about cell lines

Cell line source(s)	HEK-293F (ThermoFisher Scientific)
Authentication	None
Mycoplasma contamination	Tested free by PCR.
Commonly misidentified lines (See ICLAC register)	NA

Animals and other organisms

Policy information about studies involving animals; ARRIVE guidelines recommended for reporting animal research

Laboratory animals	Female BALB/c mice at 8-15 weeks age, a, Male db/db mice of 10-12 weeks old, C57BL/6 mice 8-20 weeks old, both male and female
Wild animals	NA
Field-collected samples	NA
Ethics oversight	Animal protocol: IACUC of the University of Chicago, protocol number 72450

Note that full information on the approval of the study protocol must also be provided in the manuscript.
